# Cerebellar hemorrhages in very preterm infants: presence, involvement of the dentate nucleus, and cerebellar hypoplasia are associated with adverse cognitive outcomes

**DOI:** 10.1007/s00330-025-11452-0

**Published:** 2025-02-20

**Authors:** Karla Drommelschmidt, Thomas Mayrhofer, Borek Foldyna, Hanna Müller, Janika Raudzus, Sophia L. Göricke, Bernd Schweiger, Selma Sirin

**Affiliations:** 1 Department of Pediatrics I, Neonatology, Pediatric Intensive Care, and Pediatric Neurology, University Hospital Essen, University of Duisburg-Essen, Essen, Germany; 2 Center for Translational Neuro- and Behavioral Sciences (cTNBS), University Medicine Essen, Essen, Germany; 3 School of Business Studies, Stralsund, University of Applied Sciences, Stralsund, Germany; 4 Cardiovascular Imaging Research Center, Department of Radiology, Massachusetts General Hospital – Harvard Medical School, Boston, USA; 5 Division of Neonatology and Department of Pediatrics, University Hospital of Tübingen, Tübingen, Germany; 6 Department of Diagnostic and Interventional Radiology and Neuroradiology, University Hospital Essen, University of Duisburg-Essen, Essen, Germany; 7 Department of Diagnostic Imaging, University Children’s Hospital Zurich, University of Zurich, Zurich, Switzerland

**Keywords:** Preterm infant, Cerebellar hemorrhage, Dentate nucleus, Magnetic resonance imaging, Brain

## Abstract

**Objective:**

Impaired cognition is a frequent complication of prematurity, closely related to patients’ outcomes. Imaging features of cerebellar hemorrhages (CBH) related to impaired cognition are not well studied. This study evaluated the relationship between cMRI-derived CBH characteristics and clinical risk factors for adverse cognition.

**Methods:**

Our analysis is threefold: (1) We included very preterm infants (2009–2018) undergoing cMRI, and compared clinical and cMRI findings between infants with and without CBH. (2) In the CBH cohort, we associated clinical and imaging findings with cognitive outcomes (Bayley Score of Infant Development at two years corrected age, impaired outcomes: < 85) using uni- and multivariable logistic regression analyses. (3) We conducted a matched pair case-control analysis (CBH vs. no CBH) matching for gestational age (GA) and supratentorial injury.

**Results:**

Among the 507 infants (52% male; mean GA 26.8 ± 2.7 weeks), 53 (10.5%) presented with CBH. Cognition was impaired in those with CBH (case-control: 88 (IQR: 75–110) vs. 105 (IQR: 90–112), *p* < 0.001), even in those with CBH < 5 mm (case-control: 95 (IQR: 77.5–115) vs. 105 (IQR: 91–113), *p* = 0.037). In infants with CBH, red-blood-cell-transfusion requirement (odds ratio (OR) 1.32, 95% CI: 1.01–1.72, *p* = 0.037), dentate nucleus involvement (OR 17.61, 95% CI: 1.83–169.83, *p* = 0.013) and moderate-to-severe cerebellar hypoplasia (OR 26.41, 95% CI: 1.11–626.21, *p* = 0.043) were independent predictors of impaired cognition. Adding dentate nucleus involvement to cerebellar hypoplasia increased the discriminatory capacity (AUC 0.85 vs. 0.71, *p* = 0.004).

**Conclusion:**

CBH (even < 5 mm) impact cognitive outcomes of very preterm infants, underlining the cerebellum’s importance for cognition. In infants with CBH, involvement of the dentate nucleus and moderate-to-severe cerebellar hypoplasia are independent structural risk factors for impaired cognition.

**Key Points:**

***Question*** *The cerebellum is important for cognition. Cerebellar hemorrhages are common in preterm infants, but the imaging features related to impaired cognition are not well studied.*

***Findings*** *Even small cerebellar hemorrhages affected cognition. Involvement of the dentate nucleus and moderate-to-severe cerebellar hypoplasia were identified as new structural risk factors for adverse cognition.*

***Clinical relevance*** *Cerebral MRI enables precise diagnosis of cerebellar hemorrhages and the detection of structural risk factors for adverse cognition like dentate nucleus involvement and cerebellar moderate-to-severe hypoplasia. This knowledge facilitates risk estimation, structured follow-up, and interventions after prematurity.*

## Introduction

Worldwide, one in ten newborns is born prematurely [1]. Despite notable progress in obstetrics and neonatology, very preterm infants (gestational age (GA) below 32 weeks) frequently suffer from neurological impairment encompassing motor, cognitive, and behavioral deficits, persisting into adulthood [2]. Notably, cognitive deficits have increased in recent years and have become a focus of interest [3–6]. Brain injuries are well-known key predictors of adverse neurological outcomes in very preterm infants [7]; in addition to supratentorial injuries, cerebellar hemorrhages (CBH) are important [8–11]. Developmentally, the cerebellum is particularly vulnerable to damage during the third trimester, which coincides with preterm birth [12]. Recently, Volpe et al underscored the impact of CBH on the cognitive outcomes of premature infants [13, 14].

Previous studies have emphasized the use of cranial magnetic resonance imaging (cMRI) in preterm infants [15, 16], as it is superior to cranial ultrasound in detecting pathologies of the posterior fossa, including CBH [7, 9, 17, 18]. CBH are frequent, with a reported incidence of up to 30% after prematurity [8], usually occurring before 27 weeks of gestation [19]. Arulkumaran et al emphasized that large cerebellar hemorrhages are associated with poor outcomes [8]. Kidokoro et al classified CBH according to size and laterality integrated into a general brain injury score [11], whereas Arulkumaran progressed toward a structural-functional correlation [8]. Therefore, further research is necessary to clarify these different approaches.

The knowledge gap primarily concerns the significance of small CBH, which are often assumed to have minor clinical relevance [20], as well as the risk factors for cognitive outcomes following CBH. There is only limited data on the effect of prematurity on the dentate nucleus [21, 22], and the consequences of hemorrhage involving the dentate nucleus on cognition are unknown despite its crucial role as a junction point connecting the cerebellum with supratentorial regions. To our knowledge, no study has evaluated different grades of cerebellar hypoplasia and their impact on cognition after prematurity. Thus, a precise anatomical-functional categorization of cerebellar damage following CBH may enhance outcome prediction, highlighting its‘ multifaceted functions [23–25].

This study assessed the impact of CBH on cognitive outcomes in a large consecutive cohort of very preterm infants over 10 years. Additionally, the study aimed to identify cerebellar structural and clinical risk factors that might negatively affect cognitive outcomes, focusing on establishing a link between cMRI findings at term-equivalent age (TEA) and cognitive function. Furthermore, we added a matched pair case-control analysis to understand the sole impact of CBH on cognition, accounting for GA and supratentorial injury.

## Methods

### Study design and patient characteristics

A retrospective observational cohort study was conducted between 01.01.2009 and 31.12.2018 of all preterm infants born < 32 + 0 weeks of gestation and treated in a large tertiary center (level III Neonatal Intensive Care Unit (NICU)). A detailed description of the patient population has been reported recently [26]. In short, clinical parameters were collected from medical records and are presented in Table 1.

**Table 1:** Neonatal characteristics of the study group, stratified by preterm infants with cerebellar hemorrhages and infants without cerebellar hemorrhages

Neonatal characteristics (mean ± SD, median [IQR], or *n* [%])	Total (*n* = 507, 100%)	No CBH (*n* = 454, 89.55%)	CBH (*n* = 53, 10.5%)	*p*-value
Weeks of gestation at birth—weeks	28.3 ± 2.3	28.5 ± 2.2	26.8 ± 2.7	< 0.001
Birthweight—g	1170.3 ± 386.5	1196.3 ± 368.3	947.3 ± 464.5	< 0.001
Birthweight < 1000 g	182 (35.9)	148 (32.6)	34 (64.2)	< 0.001
Percentile (%)	37.8 ± 23.1	38.4 ± 22.8	32.3 ± 25.0	0.101
Male sex	264 (52.1)	244 (53.7)	20 (37.7)	0.030
Delivery				0.265
Vaginal	19 (3.8)	15 (3.3)	4 (7.6)	
Primary Cesarean section	386 (76.1)	348 (76.7)	38 (71.7)	
Secondary Cesarean section	102 (20.1)	91 (20.0)	11 (20.8)	
Multiple births	144 (28.4)	132 (29.1)	12 (22.6)	0.421
PPROM –h (median + IQR)				
Median (IQR)	0 (0–3)	0 (0–4)	0 (0–0)	0.643
Mean ± SD	70.3 ± 284.4	68.2 ± 285.5	89.9 ± 276.5	0.624
SGA	62 (12.4)	53 (11.8)	9 (17.7)	0.258
Admission temperature (°C)	36.9 ± 0.6	36.9 ± 0.6	36.8 ± 0.7	0.414
Catecholamine treatment	38 (7.5)	20 (4.4)	18 (34.0)	< 0.001
APGAR 10 min	8.6 ± 0.9	8.6 ± 0.9	8.2 ± 1.1	0.005
Sepsis	161 (31.8)	132 (29.1)	29 (54.7)	< 0.001
PDA	414 (81.8)	367 (81.0)	47 (88.7)	0.193
Red-blood-cell-transfusion				
Median (IQR)	0 (0–1)	0 (0–1)	2 (0–4)	< 0.001
Mean ± SD	1.0 ± 2.2	0.8 ± 1.7	3.2 ± 4.1	< 0.001
Ventilation (invasive, d)				
Median (IQR)	0 (0–3)	0 (0–2)	7 (0–15)	< 0.001
Mean ± SD	4.2 ± 10.8	3.3 ± 10.0	11.9 ± 14.5	< 0.001

Significant: *p* < 0.05

*PDA* persistent ductus arteriosus, *PPROM* preterm premature rupture of the membranes, *SGA* small for gestational age

The inclusion criteria were: (1) in-house birth and treatment in our hospital until discharge (with readmission at TEA) or TEA, and (2) survival until TEA. Exclusion criteria were: (1) cMRI unavailable at TEA, (2) known/suspected genetic disorders, and (3) congenital infections.

The study was approved by the local Institutional Review Board and Ethics Committee of the University of Duisburg-Essen (ID: 12-4981-BO). Due to the study’s retrospective nature, the need to obtain informed consent was waived. This study was performed according to the Standards of Reporting of Diagnostic Accuracy Studies statement (STARD [27]).

### Cranial magnetic resonance imaging

A 3 Tesla scanner was used in most of the preterm infants (*n* = 400, Magnetom Skyra, Siemens Healthcare), often using an MR-compatible incubator (Lammers Medical Technology (LMT) nomag IC) with dedicated neonatal head coil as previously described [28] or on 1.5 Tesla scanners (*n* = 107, Magnetom Avanto or Magnetom Aera, Siemens Healthcare). Routine imaging was performed with the “feed and wrap” method. If sedation was necessary, we administered chloral hydrate orally (20–50 mg/kg). The routine imaging protocol contained T2-weighted turbo spin-echo (TSE) imaging (transversal), 3D T1-weighted sequence sagittal, susceptibility-weighted imaging (SWI), and diffusion-weighted imaging (DWI/DTI), as published before [28].

Two pediatric radiologists (S.S. and B.S.) with 12 and 25 years of experience analyzed the images in consensus, accompanied by a neonatologist (K.D.), blinded to clinical data. Details and results of the cMRI analysis of the cohort 2009–2018 were published [26].

**Fig. 1:**
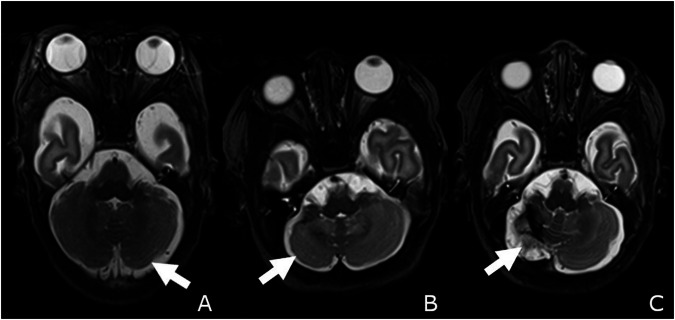
Cerebellar hypoplasia: Mild (1°, white arrow in **A**), moderate (2°, white arrow in **B**), and severe (3°, white arrow in **C**) cerebellar hypoplasia. **A** Male infant born at 26 weeks of gestation with mild cerebellar hypoplasia on the left side (1°, < 25% hypoplasia of the affected cerebellar hemisphere). **B** Male infant born at 25 weeks of gestation and moderate hypoplasia on the right side (2°, 25–50% hypoplasia of the affected cerebellar hemisphere). **C** Female infant born at 28 weeks of gestation with severe hypoplasia on the right side (3°, > 50% hypoplasia of the affected cerebellar hemisphere, all T2-weighted TSE-sequences)

For this study, infants with CBH were further evaluated regarding the size (number of bleedings), affected hemispheres (right/left/bilateral), length of the most extensive bleeding (mm), extension (< 5/≥ 5–15/> 15 mm), the extent of bleeding (total combined): cumulative length of bleeding/infant (mm), involvement of the dentate nucleus (right/left/bilateral), affected lobes (anterior/posterior, right/left/bilateral), vermis, cerebellar hypoplasia with severity grades (1° (mild): < 25%, 2° (moderate): 25–50%, 3° (severe): > 50%, right/left/bilateral), hemispheres (right/left/bilateral affected). Moderate-to-severe hypoplasia was defined as either hypoplasia 2° or 3°. In cases of unilateral hemorrhages, we graded the affected cerebellar hemisphere compared to the contralateral non-affected cerebellar hemisphere to define the grade of cerebellar hypoplasia. In the cases of bilateral cerebellar hemorrhages, we defined the grade regarding the presumed normal cerebellar hemisphere, considering the size of the osseous posterior fossa with consecutively enlarged subarachnoidal spaces in cases of cerebellar hypoplasia. We illustrated examples for the different hypoplasia grades in Fig. 1. To ensure the intraparenchymal location of the hemorrhage and to differentiate cerebellar intraparenchymal from subarachnoidal or intraventricular blood products, we correlated each SWI-findings with the corresponding T2- and 3D T1-weighted sequences and only rated them as CBH in cases with assured intraparenchymal location. CBH were classified according to Kidokoro as described before [20].

### Neurodevelopmental assessment

Preterm infants born < 32 weeks of gestation participated in a follow-up program as part of the clinical routine; Bayley Score of Infant Development (BSID II/III) was calculated at a corrected age of 24 months. The composite scores were reported as a mean with standard deviation. Cognitive and motor skills are being assessed with the BSID. We defined scores < 85 as an impaired outcome (developmental quotient > 1 SD below the mean) and ≥ 85 as a favorable outcome, according to Arulkumaran et al [8].

### Statistical analysis

Continuous variables are presented as mean ± standard deviation (SD) or median and interquartile range (IQR). Categorical variables are presented as absolute and relative frequencies. Comparisons were performed using an independent sample t-test, Wilcoxon rank-sum test for continuous variables, and Fisher’s exact test for categorical variables.

To assess the relationship of cerebellar and clinical risk factors to neurodevelopmental outcomes in CBH infants, we used univariable and multivariable logistic regression models. First, variables *p* < 0.1 in univariable analyses were identified and selected for multivariable models. These associations were further interrogated in three multivariable models: Model 1 included all selected cerebellar risk factors while Model 2 included all selected clinical factors. Model 3 included all variables that were *p* < 0.1 in Models 1 and 2. Model 3 included all significant variables from models 1 and 2. To assess whether significant variables in Model 3 are not only independent of but also incremental to each other, we compared areas under the receiver operating characteristics curve (AUC) using the Likelihood ratio test.

**Fig. 2:**
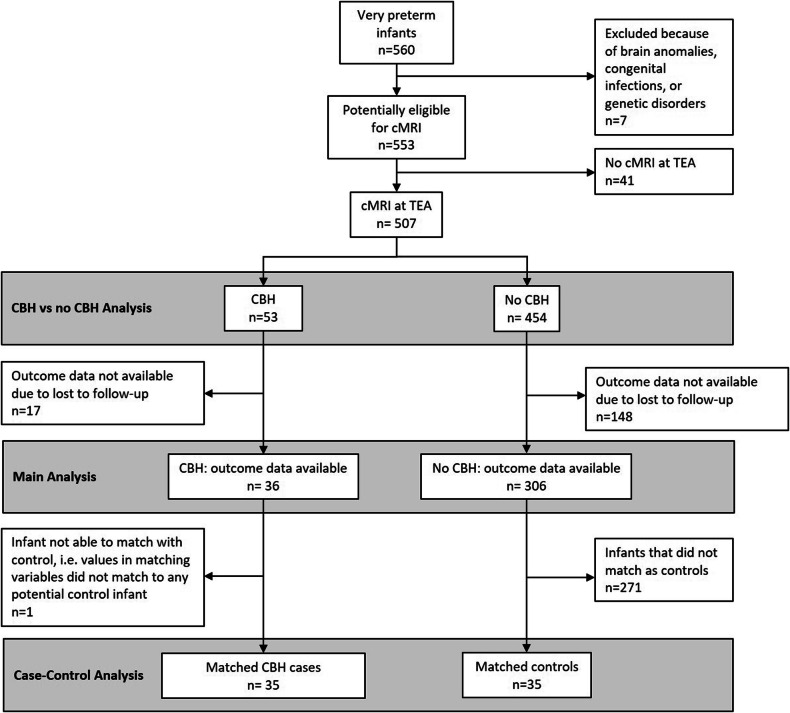
Study population

We conducted a matched pair case-control analysis. All cases with CBH and available outcomes (except one infant, see Fig. 2) were matched with controls without CBH regarding gestational age and the following supratentorial injuries: IVH I°-III°, PVHI, PVL cysts. Comparisons between matched groups were performed using a paired t-test for continuous variables, the Wilcoxon signed-rank test for continuous variables, ordinal variables, and the McNemar test for categorical (binary) variables.

Due to this study’s exploratory character, the inference was guided by a two-sided 5% false positive error rate without adjustment for multiple comparisons. All statistical analyses were performed using Stata 16.1 (StataCorp LP).

## Results

### CBH study population

The study included a large consecutive cohort of 507 preterm infants (< 32 weeks, Fig. 2) receiving cMRI at TEA (GA (Median): 40 + 1 (IQR: 0–4) weeks). The clinical characteristics are presented in Table 1. CBH were present in 10.5% of the infants (*n* = 53/507). Compared to the infants without CBH (*n* = 454), the infants with CBH had a lower GA (26.8 ± 2.7 vs. 28.5 ± 2.2 weeks, *p* < 0.001), a lower birthweight (*p* < 0.001), were more frequently female (*p* = 0.030), had a lower APGAR at 10 min (*p* = 0.005), needed more often catecholamine treatment (*p* < 0.001), suffered more frequently from sepsis (*p* < 0.001), received a higher number of red-blood-cell (RBC)-transfusions (*p* < 0.001), and were longer ventilated invasively (*p* < 0.001, Table 1).

Regarding the presence of concomitant supratentorial brain injuries (Table 2), infants with CBH suffered more frequently from intraventricular hemorrhage (IVH, 41.5% vs. 13.9%, *p* < 0.001), higher grades of ventricular dilatation (*p* = 0.005), and a higher number of brain injuries (*p* < 0.001) and severe brain injuries (*p* = 0.001).

**Table 2:** CMRI detected concomitant supratentorial brain injuries in preterm infants with cerebellar hemorrhages and infants without cerebellar hemorrhages

Brain injury (mean ± SD, median [IQR], or *n* [%])	Total (*n* = 507)	No CBH (*n* = 454)	CBH (*n* = 53)	*p*-value
IVH				< 0.001
None	422 (83.2)	391 (86.1)	31 (58.5)	
IVH I°	33 (6.5)	26 (5.7)	7 (13.2)	
IVH II°	37 (7.3)	27 (6.0)	10 (18.9)	
IVH III°	15 (3.0)	10 (2.2)	5 (9.4)	
PVHI	4 (0.8)	2 (0.4)	2 (3.8)	0.056
Ventricular dilatation				0.005
None	41 (8.1)	38 (8.4)	3 (5.7)	
Mild	341 (67.3)	314 (69.2)	27 (50.9)	
Moderate	104 (20.5)	87 (19.2)	17 (32.1)	
Severe	21 (4.1)	15 (3.3)	6 (11.3)	
cPVL				0.076
None	490 (96.7)	441 (97.1)	49 (92.5)	
Bilateral	9 (1.8)	6 (1.3)	3 (5.7)	
Unilateral	8 (1.6)	7 (1.5)	1 (1.9)	
Punctate white matter lesions				0.682
None	415 (81.9)	373 (82.2)	42 (79.3)	
Bilateral	71 (14.0)	63 (13.9)	8 (15.1)	
Unilateral	21 (4.1)	18 (4.0)	3 (5.7)	
≥ 6	50 (9.9)	46 (10.1)	4 (7.6)	0.807
≥ 10	23 (4.5)	22 (4.9)	1 (1.9)	0.495
DEHSI	490 (96.7)	437 (96.3)	53 (100)	0.239
Number of brain injuries				< 0.001
0	262 (51.7)	262 (57.7)	0 (0)	
1	152 (30.0)	135 (29.7)	17 (32.1)	
2	59 (11.6)	41 (9.0)	18 (34.0)	
3	26 (5.1)	13 (2.9)	13 (24.5)	
4	6 (1.2)	3 (0.7)	3 (5.7)	
≥ 5	2 (0.4)	0 (0)	2 (3.8)	
> 1 Brain injury	93 (18.3)	57 (12.6)	36 (67.9)	< 0.001
Severe brain injuries	61 (12.0)	34 (7.5)	27 (50.9)	< 0.001
Number of severe brain injuries				< 0.001
0	446 (88.0)	420 (92.5)	26 (49.1)	
1	45 (8.9)	25 (5.5)	20 (37.7)	
2	9 (1.8)	5 (1.1)	4 (7.6)	
3	5 (1.0)	4 (0.9)	1 (1.9)	
4	1 (0.2)	0 (0)	1 (1.9)	
≥ 5	1 (0.2)	0 (0)	1 (1.9)	
> 1 Severe brain injury	16 (3.2)	9 (2.0)	7 (13.2)	0.001

Brain injury: IVH I°-III°, PVHI, moderate and severe VD, CBH, punctate white matter lesions, cPVL

Severe brain injury: IVH III°, PVHI, CBH III° + IV°, severe VD, cPVL; significant: *p* < 0.05

*CBH* cerebellar hemorrhages, *cPVL* cystic periventricular leukomalacia, *DEHSI* diffuse excessive high signal intensity, *IVH* intraventricular hemorrhage, *PVHI* periventricular hemorrhagic infarction, *VD* ventricular dilatation

#### Size and localization of CBH

In infants with CBH, the frequency of CBH < 5 mm was high (62.3%), whereas extensive hemorrhages (> 15 mm) were less common (17.0%). In 62.3%, hemorrhage was unilaterally sited (right: *n* = 20, left: *n* = 10) and bilaterally in 37.7% of cases. The posterior lobe was affected by CBH in 84.9% (only right/left: 42.2%/20%, bilateral: 37.8%) and the anterior lobe in 41.5% (right: 40.9%, left: 45.5%, bilateral: 13.6%) of the cases. In 52.8% of the cases, only the posterior lobe was involved; in 9.4%, only the anterior lobe and in 32.1%, both lobes were affected.

**Fig. 3:**
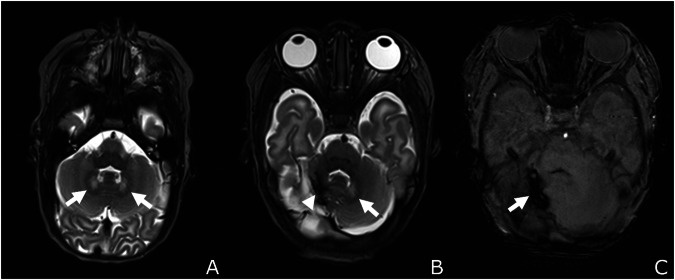
Cerebellar hemorrhage with involvement of the dentate nucleus (**B**, **C**). **A** Male preterm infant born at 27 weeks of gestation without cerebellar hemorrhage and normal appearance of the dentate nucleus on both sides (white arrows, T2-weighted sequence). **B** Female infant born at 28 weeks of gestation with a sizeable cerebellar hemorrhage and involvement of the right dentate nucleus (white arrowhead, left hemisphere: normal appearance of the dentate nucleus marked: white arrow, T2-weighted sequence) and **C** corresponding SWI-sequence (white arrow: cerebellar hemorrhage)

The dentate nucleus was involved in 49.1% of the CBH (Fig. 3), with 76.9% showing unilateral involvement (only right: 65%, only left: 35%) and 11.3% showing bilateral involvement (Table 3). Hemorrhages of the vermis were detected in 35.8% of the infants.

**Table 3:** Association between affected cerebellar structures by hemorrhage and cognitive outcomes

Affected structures by cerebellar hemorrhages	Total (*n* = 36)	Cognitive outcome < 85 (*n* = 15)	Cognitive outcome ≥ 85 (*n* = 21)	*p*-value
CBH Score (Kidokoro)				0.308
CBH I°	16 (44.4)	4 (26.7)	12 (57.1)	
CBH II°	3 (8.3)	2 (13.3)	1 (4.8)	
CBH III°	8 (22.2)	4 (26.7)	4 (19.1)	
CBH IV°	9 (25.0)	5 (33.3)	4 (19.1)	
CBH extent (total combined)				
Median (IQR)	5.5 (2–16)	6 (3–21)	4 (2–9)	0.125
Mean ± SD	10.2 ± 13.7	15.1 ± 19.3	6.7 ± 6.2	0.120
Frequency of CBH (max.)				
Median (IQR)	2 (1–3)	2 (1–4)	1 (1–2)	0.242
Mean ± SD	3.3 ± 6.9	4.9 ± 10.4	2.1 ± 2.0	0.324
CBH extent (max.)				
Median (IQR)	3 (2–11)	4 (2–15)	2 (2–4)	0.097
Mean ± SD	6.2 ± 6.3	8.5 ± 7.8	4.5 ± 4.5	0.089
CBH extent				0.142
< 5 mm	24 (72.7)	8 (57.1)	16 (84.2)	
5–15 mm	4 (12.1)	2 (14.3)	2 (10.5)	
> 15 mm	5 (15.2)	4 (28.6)	1 (5.3)	
Hemisphere				0.245
None	0 (0)	0 (0)	0 (0)	
Only right	15 (41.7)	4 (26.7)	11 (52.4)	
Only left	9 (25.0)	4 (26.7)	5 (23.8)	
Bilateral	12 (33.3)	7 (46.7)	5 (23.8)	
Dentate nucleus (yes/no) (*n*/%)	17 (47.2)	12 (80.0)	5 (23.8)	0.002
Dentate nucleus				0.003
None	19 (52.8)	3 (20.0)	16 (76.2)	
Only right	9 (25.0)	6 (40.0)	3 (14.3)	
Only left	5 (13.9)	3 (20.0)	2 (9.5)	
Bilateral	3 (8.3)	3 (20.0)	0 (0)	
Hypoplasia (*n*/%)	13 (36.1)	8 (53.3)	5 (23.8)	0.090
Hypoplasia 2° + 3°	8 (22.2)	7 (46.7)	1 (4.8)	0.005
Hypoplasia				0.106
None	23 (63.9)	7 (46.7)	16 (76.2)	
Only right	5 (13.9)	3 (20)	2 (9.5)	
Only left	5 (13.9)	2 (13.3)	3 (14.3)	
Bilateral	3 (8.3)	3 (20.0)	0 (0)	
Vermis (*n*/%)	12 (33.3)	7 (46.7)	5 (23.8)	0.175
Lobe anterior (yes/no)	14 (38.9)	9 (60.0)	5 (23.8)	0.041
Lobe anterior				0.068
None	22 (61.1)	6 (40.0)	16 (76.2)	
Only right	4 (11.1)	3 (20.0)	1 (4.8)	
Only left	8 (22.2)	4 (26.7)	4 (19.1)	
Bilateral	2 (5.6)	2 (13.3)	0 (0)	
Lobe posterior (yes/no)	34 (94.4)	14 (93.3)	20 (95.2)	1.000
Lobe posterior				0.332
None	2 (5.6)	1 (6.7)	1 (4.8)	
Only right	14 (38.9)	4 (26.7)	10 (47.6)	
Only left	9 (25.0)	3 (20.0)	6 (28.6)	
Bilateral	11 (30.6)	7 (46.7)	4 (19.1)	

Significant: *p* < 0.05

*CBH* cerebellar hemorrhages

#### Cerebellar hypoplasia

Cerebellar hypoplasia was present in 34.0% of the preterm infants (right hemisphere: 22.6%, left hemisphere: 18.9%, bilaterally: 5.7%). Mild hypoplasia (1°) was seen in 31.8%, moderate (2°) in 36.4%, and severe hypoplasia (3°) in 31.8% (Fig. 1).

## Cognitive outcomes

### Matched pair case-control study

**Fig. 4:**
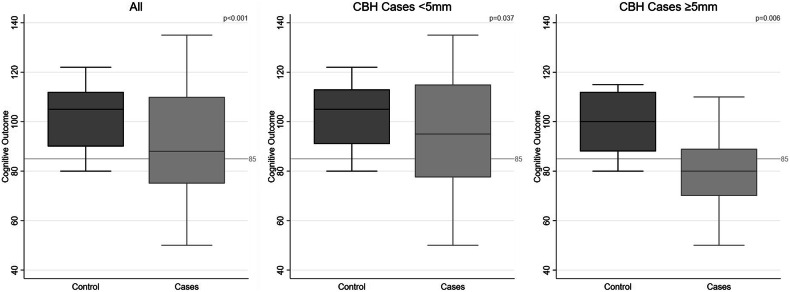
Case-control study: association between infants with cerebellar hemorrhage (CBH) vs controls without CBH and stratified for bleeding size: infants with any cerebellar hemorrhages vs controls, infants with cerebellar hemorrhages < 5 mm, and infants with cerebellar hemorrhages ≥ 5 mm. CBH, cerebellar hemorrhages; significant: *p* < 0.05

After matching with controls for GA (27.0 ± 0.4 vs. 27.3 ± 0.4 weeks) and supratentorial brain injuries, significantly lower cognitive scores for the infants with CBH compared to the matched controls (88 (IQR: 75–110) vs. 105 (IQR: 90–112), *p* < 0.001) were detected. In the sub-analysis stratified by bleeding size, lower cognitive scores were found not only in infants with large CBH (CBH ≥ 5 mm, 80 (IQR: 70–89) vs. 100 (IQR: 88–112), *p* = 0.006) but also for hemorrhages < 5 mm (95 (IQR: 77.5–115) vs. 105 (IQR: 91–113), *p* = 0.037, Fig. 4, with an overlap of the IQR).

### Analysis of prognostic factors on outcomes within the CBH cohort

#### Clinical risk factors

The following clinical parameters were associated with impaired cognition (cognitive score < 85): GA (25.5 ± 2.2 vs. 27.4 ± 2.8 weeks, *p* = 0.034), birthweight (713 ± 336 vs. 1074 ± 587 g, *p* = 0.026), catecholamine treatment (53.3% vs. 14.3%, *p* = 0.025), RBC-transfusions (6.3 ± 4.9 vs. 2.0 ± 3.6, *p* = 0.008), invasive ventilation (15 (6–35) vs. 2 (0–12) days, *p* = 0.023, Supplementary Table 1).

#### CMRI risk factors

We found no significant difference between CBH affecting the right or left hemisphere (*p* = 0.245). CBH in infants involving the anterior lobe were associated with impaired cognition (*p* = 0.041).

**Fig. 5:**
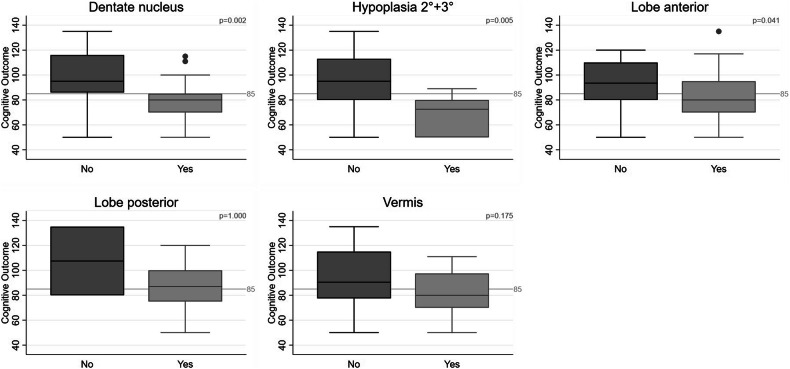
Association between affected cerebellar structures and cognitive outcomes. The dentate nucleus (*p* = 0.002), moderate + severe cerebellar hypoplasia (*p* = 0.005), lobe anterior (*p* = 0.041), lobe posterior (*p* = 1.000), and the vermis (*p* = 0.175) are shown, significant: *p* < 0.05

Dentate nucleus involvement was associated with lower cognitive outcomes (80.0% scored < 85, *p* = 0.002, Fig. 5). The localization of the affected dentate nucleus impacted cognition (CO < 85/ ≥ 85: only right: 40%/14.3%, only left: 20%/9.5%, bilateral: 20%/0%, *p* = 0.003). Adverse cognitive outcomes were more frequent in the presence of moderate-to-severe hypoplasia (46.7%/4.8%, *p* = 0.005, Fig. 5). Impaired cognition was detected in 46.7% of the infants with affected vermis (no involvement: 23.8%, *p* = 0.175, Table 3, Fig. 5).

### Risk factors for lower cognitive outcomes in CBH infants

#### Clinical risk factors

The need for RBC-transfusions was associated with a higher risk for impaired cognitive outcomes (odds ratio (OR): 1.32, CI: 1.01–1.72, *p* = 0.087, Table 4, Model 1), although the association did not reach statistical significance.

**Table 4:** Association between affected cerebellar structures after hemorrhage, clinical factors, and adverse cognitive outcomes

	Model 1			Model 2			Model 3		
	Odds	95% CI	*p*-value	Odds	95% CI	*p*-value	Odds	95% CI	*p*-value
Weeks of gestation at birth—weeks	1.05	0.55–2.02	0.878						
Birthweight—g	1.00	1.00–1.00	0.372						
Catecholamine treatment	2.65	0.40–17.40	0.311						
Red-blood-cell-transfusion (continuous score values)	1.64	0.93–2.90	0.087				1.32	1.01–1.72	0.037
Ventilation (invasive) (d)	0.90	0.79–1.03	0.142						
Dentate nucleus				10.45	1.25–87.28	0.030	17.61	1.83–169.83	0.013
Hypoplasia 2° + 3°				23.95	1.15–499.03	0.040	26.41	1.11–626.21	0.043
Lobe anterior				2.13	0.27–16.90	0.476			
CBH Score (Kidokoro)									
CBH I°				ref					
CBH II°				1.47	0.07–32.30	0.807			
CBH III°				1.10	0.10–12.55	0.939			
CBH IV°				0.26	0.01–5.84	0.398			

Association between affected cerebellar structures after hemorrhage, clinical factors, and adverse cognitive outcome (Bayley Score of Infant Development, cognitive outcome: < 85). Logistic Regression for cognitive outcome. Model 1 included cerebellar risk factors statistically significant in univariable analyses; Model 2 included all significant clinical factors. Model 3 included all significant variables from models 1 and 2; significant: *p* < 0.1

*CBH* cerebellar hemorrhages

#### Affected cerebellar structures as risk factors

The dentate nucleus and moderate-to-severe hypoplasia emerged as independent risk factors associated with adverse cognitive outcomes. In the case of dentate nucleus involvement, the risk of adverse outcomes was 10.45 times higher (CI: 1.25–87.28, *p* = 0.03) compared to infants without involvement. Moderate-to-severe hypoplasia was associated with a higher risk for adverse cognitive outcomes (OR: 23.95, CI: 1.15–499.03, *p* = 0.04). No significant correlation was found between the Kidokoro Classification grades and cognition (Table 4, Model 2).

#### ROC analysis

**Fig. 6:**
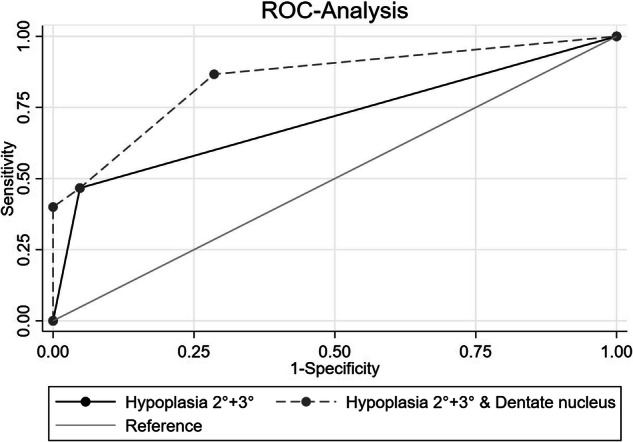
ROC-curve analysis of the most influential risk predictors within cerebellar risk factors. AUC: hypoplasia 2° + 3°: AUC = 0.71 vs. hypoplasia 2° + 3° and affected dentate nucleus: AUC = 0.85, *p* = 0.004

In terms of ROC analysis, moderate-to-severe hypoplasia demonstrated an AUC of 0.71. Incorporating dentate nucleus involvement in the model led to a significant increase in the AUC to 0.85, suggesting an incremental impact on cognitive outcomes (*p* = 0.004, Fig. 6).

#### Among the risk factors influencing cognitive outcomes after CBH

Model 3, which encompassed all significant variables from models 1 and 2, revealed that the involvement of the dentate nucleus was linked to a significantly higher risk of impaired cognitive outcomes (OR: 17.61, CI: 1.83–169.83, *p* = 0.013). Additionally, moderate-to-severe hypoplasia showed an odds ratio of 26.41 (CI: 1.11–626.21, *p* = 0.043). Furthermore, the need for RBC-transfusions increased the risk of poor outcomes by 1.32 per transfusion (Table 4; univariable analyses: Supplementary Table 2).

## Discussion

In this study on a large cohort of 507 consecutively born very preterm infants (< 32 weeks of gestation) over 10 years, we showed that cerebellar hemorrhages significantly affect cognitive outcomes following prematurity. Large and small CBH (< 5 mm) were associated with impaired cognitive outcomes compared to controls. CBH involving the dentate nucleus, a crucial connection point between the cerebellum and the cerebrum, have a significant impact on cognition in high-risk infants. This impact has not been published yet. Additionally, we identified moderate-to-severe cerebellar hypoplasia as an independent risk factor for adverse cognition. Our findings underscore the impact of the cerebellum on cognition, corroborating previous assertions by J.J. Volpe [13].

CBH are common in preterm infants, affecting approximately one in every ten infants in our study, consistent with previous studies [8, 11, 29]. We observed that CBH were more prevalent in immature and critically ill infants [3, 30]. In addition to established clinical risk factors for CBH, such as sepsis and conditions impacting the infant’s hemodynamics—like the administration of catecholamines for arterial hypotension and PDA [9, 29, 31, 32]—our study revealed the need for RBC-transfusions (also inflicting hemodynamics) as a novel risk factor for CBH. We found that the need for RBC-transfusions is an independent and significant predictor of the cognitive outcomes of very preterm infants. We are unable to explain why female sex was identified as a risk factor for CBH in our cohort. However, Vesoulis et al described male sex as a lower risk factor [30]. Further research is necessary to clarify this aspect. In conclusion, CBH are commonly observed in preterm infants, particularly affecting infants who are more immature and critically ill.

Concomitant brain injuries are frequent in infants with CBH, making it challenging to distinguish between direct, bleeding-induced effects of cerebellar injury and indirect effects of supratentorial injuries due to cerebro-cerebellar connections affecting the cerebellum [23, 33]. In our cohort, supratentorial brain injuries were significantly more frequent in infants with CBH compared to those without CBH. We conducted a matched pair case-control study to control for indirect effects due to supratentorial injuries and clarify the direct impact of cerebellar injury after CBH on cognition. We demonstrated that CBH, even small hemorrhages < 5 mm, were negatively associated with cognitive outcomes. While previous studies also found an effect of extensive cerebellar hemorrhages on cognition [13, 16], the influence of small and punctate hemorrhages remains inconclusive, often presumed to be minor [9, 20]. Our findings underline the importance of thoroughly evaluating the cerebellum after prematurity to detect even small hemorrhages. The presence of CBH, particularly small ones and those involving the dentate nucleus, is commonly missed or may not be detectable in cranial ultrasound [18, 28, 33] and cMRI without hemorrhage-sensitive sequences [8, 18]. These findings emphasize the importance of cMRI with hemorrhage-sensitive sequences like SWI for evaluation and outcome prediction.

This is, to our knowledge, the first study identifying the involvement of the dentate nucleus as an essential risk factor for adverse cognitive outcomes in preterm infants with CBH. The dentate nucleus is a crucial structure within the cerebro-cerebellar circuit, serving as an important functional junction point connecting the cerebellum and the thalamus [34]. Functional MRI studies have demonstrated the activation of the dentate nucleus during cognitive processing, confirming its important role in cognition [34, 35]. It is published that prematurity itself induces alterations [4, 31] in microstructure, as detected by cMRI [22], and modifies the function of deep cerebellar nuclei, such as dentate nuclei, following a NICU stay [36]. However, the impact of dentate nucleus damage on cognition in preterm infants remains elusive and has not been previously published. Detecting dentate nucleus damage via cMRI as a prognostic indicator underscores the importance of a thorough evaluation of the anatomical topography of the hemorrhages to predict the functional outcomes of preterm infants. Our findings indicate that it might be insufficient to merely describe the size of the hemorrhages and the affected hemisphere (both not significantly associated with cognitive outcomes in our study), as done in scoring systems [11]. In conclusion, the involvement of the dentate nucleus emerged as a critical prognostic factor for adverse cognitive outcomes in infants with CBH, which is attributed to its important role in cerebro-cerebellar functional connectivity and cognition.

Posthemorrhagic moderate-to-severe cerebellar hypoplasia was another risk factor for impaired cognition in our cohort. Hypoplasia following CBH contributes to adverse cognitive outcomes due to the mechanical damage of bleeding components, such as iron radicals, to brain tissue [6, 14, 24]. With the presence of moderate-to-severe hypoplasia, the risk for adverse cognitive outcomes increased enormously (OR: 26.41) with a high discriminatory value (AUC 0.71); the additional involvement of the dentate nucleus further increased the area under the receiver operating characteristics curve to 0.85. We could not prove that involvement of the vermis is a risk factor for adverse cognition, possibly due to the low number of cases. Involvement of the anterior lobe was associated with adverse cognitive outcomes despite cognition being primarily attributed to the posterior lobe [15]. However, the involvement of the anterior lobe did not emerge as an independent risk factor in the logistic regression analysis. Further studies are needed to elucidate these aspects.

Our study has several limitations to acknowledge. First, we conducted an exploratory analysis without adjustment for multiple comparisons, utilizing data from a retrospective single-center design. The associations observed in our study should be interpreted cautiously and require further validation. Moreover, outcomes assessment was unavailable in 17 infants with CBH, and further prospective studies are needed. Additional research is essential to explore various aspects and subgroup analyses of cognitive outcomes in infants with CBH, warranting more comprehensive long-term studies.

We showed a significant association between CBH, even small hemorrhages < 5 mm, and adverse cognitive outcomes. Our study is the first to describe the crucial influence of dentate nucleus damage and moderate-to-severe hypoplasia as important risk factors for impaired cognition following CBH. This highlights the importance of routine cMRI after prematurity with integrated hemorrhage-sensitive sequences and precise anatomical-structural radiological evaluation to obtain early insights into the cognitive outcomes of high-risk preterm infants.

## Supplementary information

ELECTRONIC SUPPLEMENTARY MATERIAL

## Abbreviations

BSID: Bayley Score of Infant Development
CBH: Cerebellar hemorrhages
cMRI: Cerebral magnetic resonance imaging
cPVL: Cystic periventricular leucomalacia
DEHSI: Diffuse excessive high signal intensity
GA: Gestational age
IVH: Intraventricular hemorrhage
NICU: Neonatal intensive care unit
PDA: Persistent ductus arteriosus
PPROM: Preterm premature rupture of membranes
PVHI: Periventricular hemorrhagic infarction
RBC-transfusions: Red-blood-cell-transfusions
SGA: Small for gestational age
SWI: Susceptibility-weighted imaging
TEA: Term-equivalent age—the regular time point at which the infant would have been born had it not been prematurely delivered
