# Supplementary material for: Cerebellar hemorrhages in very preterm infants: presence, involvement of the dentate nucleus, and cerebellar hypoplasia are associated with adverse cognitive outcomes
Source: Eur Radiol. 2025 Feb 20;35(8):4747–58. doi: 10.1007/s00330-025-11452-0 (PMC12226700; doi:10.1007/s00330-025-11452-0)
Supplement: Supplementary file 1 — ELECTRONIC SUPPLEMENTARY MATERIAL [file 330_2025_11452_MOESM1_ESM.pdf]

Cerebellar Hemorrhages in Very Preterm Infants: Presence, Involvement of the Dentate Nucleus, and Cerebellar Hypoplasia Are Associated with Adverse Cognitive Outcomes

ELECTRONIC SUPPLEMENTARY MATERIAL

Table 1 suppl.: Association between neonatal characteristics and cognitive outcomes

Neonatal characteristics (mean±SD, median (IQR), or n (%))	CO<85 (n=15)	CO≥85 (n=21)	p
Weeks of gestation at birth –weeks	25.5±2.2	27.4±2.8	0.034
Birthweight –g	713±336	1074±587	0.026
Birthweight <1000 g	13(86.7)	13(61.9)	0.142
Percentile (%)	23.6±21.8	40.4±29.0	0.059
Male sex	3(20.0)	8(38.1)	0.295
Delivery			0.488
Vaginal	0(0.0)	3(14.3)	
Primary Cesarean section	11(73.3)	13(61.9)	
Secondary Cesarean section	4(26.7)	5(23.8)	
Multiple birth	5(33.3)	4(19.1)	0.443
PPROM –h	0 (0 – 0)	0 (0 – 12)	0.419
SGA	5(33.3)	2(10.0)	0.112
Admission temperature (°C)	36.6±0.7	36.9±0.5	0.205
Catecholamine treatment	8(53.3)	3(14.3)	0.025
APGAR 10 minutes	8.3±1.0	8.4±0.8	0.710
Sepsis	9(60.0)	12(57.1)	1.000
Red-blood-cell-transfusion (continuous score values)	6.3±4.9	2.0±3.6	0.008
Ventilation (invasive) (d)	15 (6 – 35)	2 (0 – 12)	0.023
PDA	12(80.0)	20(95.2)	0.287

PDA: persistent ductus arteriosus, *PPROM:* preterm premature rupture of the membranes, *SGA:* small for gestational age, significant: $p<0.05$.

Table 2 suppl.: univariable analysis for logistic regression for cognitive outcome

	Odds	CI95	P value
Weeks of gestation at birth –weeks	0.75	0.56 – 1.00	0.049
Birthweight –g	1.00	1.00 – 1.00	0.058
SGA	0.22	0.04 – 1.36	0.104
Admission temperature (°C)	0.42	0.12 – 1.51	0.183
Catecholamine treatment	6.86	1.40 – 33.57	0.018
APGAR 10 minutes	0.85	0.39 – 1.87	0.691
Sepsis	1.13	0.29 – 4.33	0.864
Red-blood-cell transfusion	1.30	1.04 – 1.61	0.019
Ventilation (invasive) (d)	1.04	0.99 – 1.09	0.092
PDA	0.20	0.02-2.15	0.184
Hemisphere			
Only right	Ref		
Only left	2.20	0.38 – 12.57	0.375
bilateral	3.85	0.76 – 19.47	0.103
Dentate nucleus(yes/no) (n/%)	12.80	2.55 – 64.37	0.002
Dentate nucleus			
None	Ref		
Only right	10.67	1.67 – 68.18	0.012
Only left	8.00	0.91 – 70.27	0.061
bilateral	1.00	----	----
Hypoplasia (n/%)	3.66	0.88 – 15.24	0.075
Hypoplasia 2°+3°	17.50	1.84 – 166.04	0.013
Hypoplasia			
None	Ref		
Only right	3.43	0.47 – 25.27	0.227
Only left	1.52	0.21 – 11.23	0.679
bilateral	1	----	----
Vermis (n/%)	2.80	0.67 – 11.67	0.157
Lobe anterior (yes/no)	4.80	1.14 – 20.27	0.033
Lobe anterior			
None	Ref		
Only right	8.00	0.69 – 92.70	0.096
Only left	2.67	0.50 – 14.22	0.251
bilateral	1	----	----
Lobe posterior (yes/no)	.70	0.04 – 12.16	.807
Lobe posterior			
None	Ref		
Only right	0.40	0.02 – 8.07	0.550
Only left	0.50	0.02 – 11.09	0.661
bilateral	1.75	0.08 – 36.29	0.718

PDA: persistent ductus arteriosus, *SGA:* small for gestational age, significant: $p<0.05$

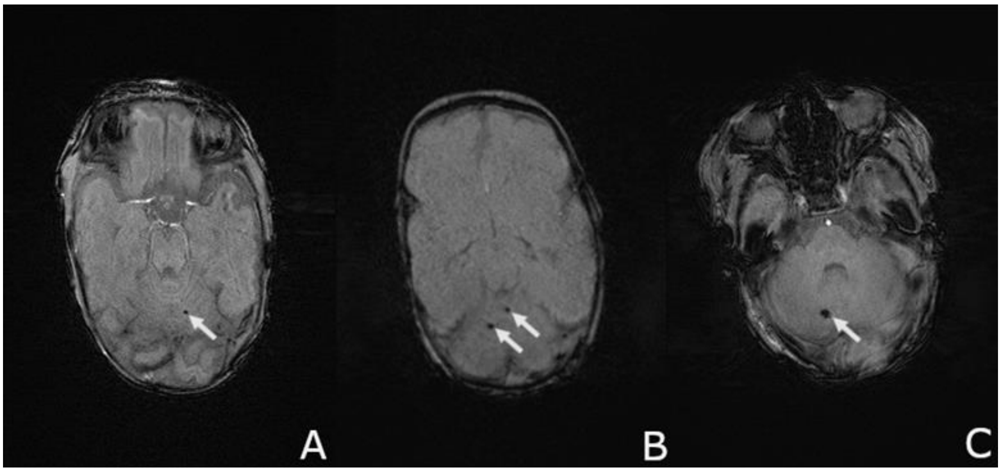

Figure 1 suppl.: Examples of cerebellar hemorrhages <5mm (white arrows, A-C: SWI sequences) in three different preterm infants.
